# Health Risks, Perceptions, and Self-Care Patterns: A Comparative Study Between Older and Younger Filipinos

**DOI:** 10.1093/geroni/igab046.569

**Published:** 2021-12-17

**Authors:** Julienne Ivan Soberano, Marysol Caciata, Jo Leah Flores, Erwin William Leyva, Mary Abigail Hernandez, Josefina Tuazon, Lorraine Evangelista

**Affiliations:** 1 University of the Philippines, Mandaluyong City, National Capital Region, Philippines; 2 VA Long Beach Healthcare System, VA Long Beach Healthcare System, California, United States; 3 University of the Philippines, University of the Philippines, National Capital Region, Philippines; 4 University of Texas Medical Branch School of Nursing, University of Texas Medical Branch School of Nursing, Texas, United States

## Abstract

Worldwide trends in health risks, lifestyle behaviors, health perceptions, and health-seeking patterns suggest alarming disparities among individuals from low- and middle-income countries; particularly for older individuals (≥ 60 years). This study aims to compare health risks, perceptions, lifestyle behaviors, and health-seeking patterns between younger (< 60 years) and older (≥ 60 years) Filipinos from rural communities in the Philippines; and assess relationships between demographic, health risks and perceptions, and lifestyle behaviors to bolster health promotion efforts. A comparative cross-sectional study was employed with 863 younger and 427 older Filipinos. Results show that older participants were more likely to be single/widowed and had ≤ high school education. Older participants had higher rates of hypertension, dyslipidemia, diabetes, and depression but were more likely to report higher quality of life, ≥ 150 minutes of physical activity per week, ≥ 5 servings of fruits and vegetable per day, more difficulty falling asleep, report seeing a physician regularly, going to the community health center when sick, and attend stress management classes compared to their younger counterparts (all p’s < .001). There were no differences in rates of obesity, self-medication, and use of integrative health. Older age was associated with higher risks, improved health perceptions, healthier lifestyle behaviors, and better health-seeking patterns. Our data suggest that health risks are higher in older individuals but risky lifestyle behaviors were higher in younger individuals and suggest the need to design separate health promotion interventions that target the unique needs of older and younger Filipinos from rural communities.

